# Filling in the Gaps: Artistic License in Education and Outreach

**DOI:** 10.1371/journal.pbio.0050308

**Published:** 2007-12-04

**Authors:** David S Goodsell, Graham T Johnson

## Abstract

Conscious and careful application of artistic license is an essential part of the creation of scientific illustrations for research, education, and science outreach.

When called upon to share our scientific results with the world, scientists are faced with a challenge: we must select appropriate representations to express our results clearly and unambiguously. At one extreme, a primary researcher must make many decisions when creating a technical report: Will a line graph work better than a bar graph, pie chart, or table of numbers? Is color needed to distinguish different features? At the opposite extreme, scientific illustrators are often called upon to assemble a speculative picture that integrates what is known within the context of what is not. As you might imagine, this process involves considerable artistic license—the distortion of fact in service of a purpose—and the amount of license considered acceptable depends highly on both the subject and the audience.

Scientific illustrators, when we are lucky, not only depict current scientific knowledge, but also contribute to hypotheses as they form.

Scientific illustrators, when we are lucky, not only depict current scientific knowledge, but also contribute to hypotheses as they form. This is particularly apparent in the creation of imagery for education and outreach. Often, there exists firm scientific evidence for a (hopefully large) portion of the subject and hypotheses and speculation for the pieces that are missing. While the bulk of scientific literature contributes piecemeal to ultimately convey this missing imagery, illustrators are called upon to, as Edward Tufte suggested in a lecture given at the 1991 meeting of the Molecular Graphics Society, “show the real world, not lines and numbers,” as they strive “to create visual solutions for visual problems.” Thus, when employed during the formative stages of a subject's understanding, illustrators can help to assemble, convey, and translate information. They present this information visually, often with great liberty, but liberty that aids in peer-to-peer communication, education, and outreach, and sometimes helps to suggest new avenues of research where seeing the data represented in a new manner, in context, or simply visually, helps the researcher formulate ideas.

## Acceptable Limits

Everyone, from primary research scientists to scientific illustrators, makes decisions about the information that will be included—and more importantly, the information that will be excluded—from their figures. If you look through the technical reports here in *PLoS Biology*, you will see a wide variety of illustration styles and types, but very little artistic license. Technical publications typically demand a tight connection between illustration and data, since the illustrations often serve as the primary evidence supporting the claims of the publication. In general, a direct algorithmic chain of methods must link the data to the illustration. No tweaking by hand is allowed: for instance, it is unethical to darken a band on a gel, edit out a few stars on an astronomical photograph, or add a missing bone to an illustration of a paleontological specimen. Illustrators must adhere to a stringent set of rules, so that readers will be able to trust that the drawing is an accurate reflection of the scientist's data. Global manipulations are often used, for example, to enhance the comprehensibility of scientific images. These may include use of false color schemes, optimization of contrast and hues, and cropping for emphasis. The goal of applying any of these techniques is to make a clearer image without unduly distorting the presentation of the data.

In educational settings, however, artistic license is more acceptable and often essential to provide a more general view of a scientific concept and to excite interest. That missing bone on the right foot of a dinosaur may be augmented by a mirrored version from the left foot to complete the skeleton. An unknown protein may be replaced by a distant homologue, or individual proteins may be presented as symbolic representations (think of the Y-shaped objects often used to depict antibodies) or random-shaped blobs. These simplified, iconic representations are designed to read quickly as “protein,” unlike detailed graphic elements or text that slow the reader and require explanation. For the general reader, even more artistic license is required, whether for educational materials that seek to cover an entire field or for popular magazines that may build an entire article around a new hypothesis. This can be taken to excess, however: for instance, some of the popular imagery representing nanotechnology is more science fiction than science.

## Tricks of the Trade

**Figure 1:**
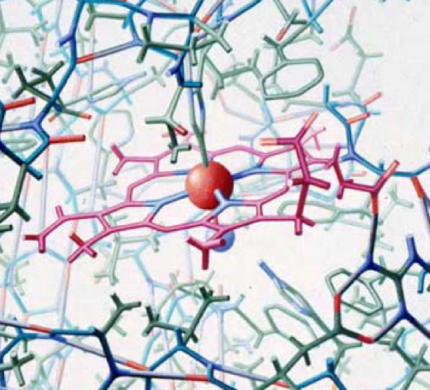
Detail of “Sperm Whale Myoglobin“ by Irving Geis, Showing the Heme Group and Iron Ion Geis used many artistic tricks to make this complex figure more comprehensible. This included the use of depth cueing and scaling of bonds to enhance the three-dimensionality of the image so that distant portions are lighter, smaller, and less prominent. Each bond is also carefully rendered so that the shading gives visual clues to its orientation in space. In addition, Geis made small adjustments to resolve any unfortunate overlaps—a process he called “selective lying.” The image was originally published in December 1961 to illustrate Sir John Kendrew's *Scientific American* article “The Three-Dimensional Structure of a Protein.” The painting is now part of the Irving Geis Collection at the Howard Hughes Medical Institute.

The pioneering molecular illustrator Irving Geis, when speaking of his groundbreaking illustrations of myoglobin and lysozyme for *Scientific American* (Figure 1), mentioned that “selective lying” was essential. Protein molecules are so complex that he needed to rely on all of the tricks of the trade, including depth cuing and careful shading—methods that are completely true to the underlying structure. But the pictures were still too confusing, due to unfortunate overlapping of portions of the structure, so he shifted portions in the front and rear slightly, resolving these overlaps and making the threedimensional relationships more clear. Today, scientific artists employ a number of similar artistic techniques to tackle the most challenging subjects. These techniques can be generally classified as filling in gaps, selective disclosure, and distortion. 

At the most basic level, artistic license is often used to fill in knowledge gaps. We never have the complete story in science, but the artist needs to create a convincing picture, so decisions have to be made about missing pieces and how to render them. Solutions may be as simple as drawing a dotted line to connect two protein domains of known structure or as complex as creating an artistic rendition of an accretion disk around a black hole.

Sometimes leaving out information can facilitate understanding. Through selective disclosure, which is similar in character to reductive approaches used in science, scientific illustrators can simplify a topic by stripping away all distracting information to focus attention on the subject at hand. For instance, most molecular illustrations are created using structural data from purified molecules, studied and depicted far from their normal cellular environment, often as an isolated individual. The illustrations presented here, although appearing more naturalistic, still omit most of the molecules in the cellular environment. Most molecular illustrations also depict only a subset of the atoms, for example, highlighting the active site, the backbone, or the surface characteristics.

**Figure 2:**
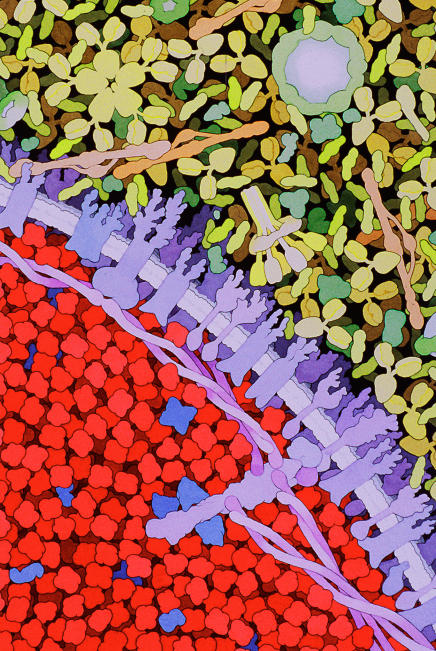
Cross Section through a Red Blood Cell The blood serum is shown at the top, the cell membrane crosses through the center, and the interior of the cell, packed with hemoglobin, is at the bottom. This illustration was created by David Goodsell for use in an online educational resource at the undergraduate college level.

**Figure 3:**
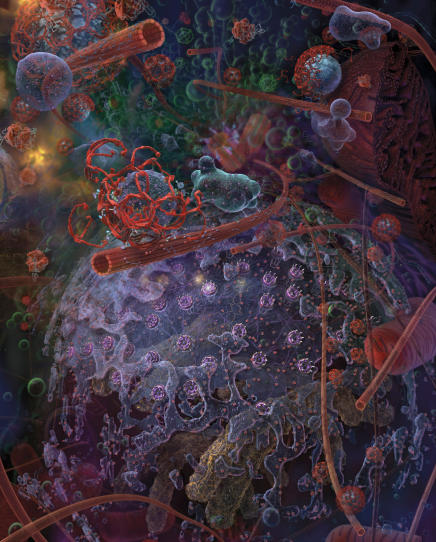
Cell Signaling This is a detail cropped from a 36-inch-tall painting created by Graham Johnson for the textbook *Cell Biology* by Tom Pollard and Bill Earnshaw (W. B Saunders-Elsevier 2007). The painting depicts the process of cell signaling, starting from an initiating signal at a cell surface receptor (not shown here) and ending with mitosis. Details along the chain of events were used as chapter openers in the book, and the entire painting was presented as a poster.

Purposeful distortion of a subject is sometimes required to compensate for the fact that an illustration cannot capture actual spatial and temporal scales. Geis used a very mild distortion to enhance the three-dimensionality of his molecules, and the molecules in Figure 2 are drawn at the same scale throughout the image, allowing easy comparison of sizes and shapes. However, scientists often explore subjects with large spatial and temporal scale ranges, and artists are called upon to make these clear. For instance, the illustration in Figure 3, described in more detail below, uses a heightened perspective distortion to display objects over several orders of magnitude in scale, and compresses processes that occur over a wide range of time scales.

## Depicting the Cellular Environment

Artistic license has played a central role in our own work of depicting the cellular mesoscale (the range that exists between the micrometer and nanometer scales). This is an interesting scale to study, since it is virtually invisible to experiment, but illustrations of molecules in cells are essential for understanding and teaching concepts in cell biology. Currently, these types of illustrations must be synthesized from diverse data at higher and lower scale levels: from atomic structures of purified molecules, from biochemical data on molecular concentrations and locations, and from microscopic ultrastructural data. Since these are complex subjects supported by abundant data, but still with many missing or as yet unimagined parts, we must use all the tricks of our trade.

Figure 2 shows a cross section through a red blood cell, along with some blood serum at the top. The goal of this picture is naturalistic: it attempts to show what the viewer might see if this small portion of the cell were magnified to a scale large enough to make individual macromolecules visible. With this subject, major license is taken with the uniform depiction. The style gives the viewer no information on the level of experimental support for each component. This approach is essential, however, to provide the natural feeling.

Everyone, from primary research scientists to scientific illustrators, makes decisions about the information that will be included—and more importantly, the information that will be excluded—from their figures.

Many other subtle tricks are used to increase the comprehensibility of this complex scene. Simple shapes are used for the molecules, allowing the eye to focus on the whole scene rather than getting caught up in all the details. The colors (except perhaps for the hemoglobin) are completely arbitrary and are chosen solely for aesthetic appeal. Looking closely, you can also see that the orientation of each molecule is not realistic. Notice that all of the antibodies are depicted in the plane of the page, so that the Y-shape is apparent, and none of the long fibrinogen molecules stick up out of the plane of the page. If completely general orientations were used, the molecules would not be nearly as recognizable.

Figure 3 shows a cross section through a eukaryotic cell, highlighting an important signaling process. The goal of this picture, created as a supplement for a new cell biology textbook, is to excite interest in science, both by providing enough detail to intrigue researchers and by couching this detail in the context of its broader biological importance to attract more general audiences. The image depicts wide ranges of both spatial and temporal scales, which proved challenging to combine in a single poster.

Through selective disclosure, which is similar in character to reductive approaches used in science, scientific illustrators can simplify a topic by stripping away all distracting information to focus attention on the subject at hand.

The illustration contains many players, with varied levels of experimental support. It starts with an initiating signal stored as information in a particular molecular conformation, transferred from the surface of the cell into the cytoplasm over a range of seconds and a distance of nanometers. Extreme foreshortening and time contraction are employed to fit everything onto a single page as we follow the signal into the nucleus, where it triggers mitosis. The image purposefully hides details, invisible from such a distance, to focus attention on the outcome of the tale, where the cell ultimately forms structures—chromosomes—on a scale many orders of magnitude larger than the initiating signaling molecule and at a time several hours later.

Many liberties were taken to make this panoramic image more comprehensible. For instance, microtubules are truncated rather than sliced to emphasize the helix seam, and microtubule-associated proteins are roughly sketched onto microtubules to imply that we know the proteins exist but still lack knowledge of their structure, diversity, scale, or propensity for binding. Throughout the scene, proteins not directly involved are subdued to near invisibility, and a highlighting glow directs attention to each step in the story. The clathrin protein, seen as geodesic structures surrounding lipid vesicles in the painting, proved to be an exciting research problem as well as an artistic challenge. The structure currently available in the Protein Data Bank (http://www.pdb.org/) is a small form with 36 vertices, so an adjustable symmetry algorithm was developed to expand the triskelion components into numerous larger cages, based on cage structures observed by lower resolution electron microscopic studies. The artist compacted time to include the initiating signal and the resulting cellular event (chromosomes forming in mitosis, depicted as squiggly orange Xs at the bottom right of the page) that occurs hours later, in a single image.

## Granting License

For a scientific illustrator, the application of artistic license is perhaps the most exciting aspect of the work. It provides a tension that keeps every project interesting and challenging. For example, in educational and outreach settings, the illustrator may be caught in a struggle between a scientist, who seeks to reduce the content to a narrow subject with firm experimental support, and an editor, who pushes the project to larger topics with more hypothetical elements and broader appeal. The challenge is to balance these needs in pictorial form.

This tension is particularly strong in molecular biology, where the amount of knowledge is growing rapidly and most of the major molecular players have been characterized. Atomic structures are now available for ribosomes, polymerases, microtubules, and all manner of molecular machines, and electron micrograph reconstructions are available for even larger assemblies, such as flagellar motors and actomyosin. Given the increasing volume of structural data that are currently available, and the widely expanding knowledge base of molecular anatomy and physiology that builds on these data, the allowable range of artistic license is steadily decreasing.

As communicators who make science accessible to colleagues, students, and the public, we must define an acceptable licensing threshold that will allow us to create evocative pictures, but will still leash us enough to avoid polluting the literature (both scientific and popular) with deceptive imagery. This is particularly important for scientists, since pictorial errors in primary scientific publications, which are often persistent sources of information, may be propagated for decades in educational and outreach publications. Thus, when we go to generate a figure for a paper, we can freely exercise our aesthetic sense on colors and viewpoints, but pictures of icosahedral viruses must have icosahedral symmetry—and images of B-DNA must be right-handed.

